# Stop, Take a Breath, and Search for Joy

**DOI:** 10.31486/toj.20.5015

**Published:** 2020

**Authors:** Ronald G. Amedee

**Affiliations:** Head of Clinical School and Professor, The University of Queensland Faculty of Medicine, Ochsner Clinical School, New Orleans, LA; Editor-in-Chief, *Ochsner Journal*

This year has certainly been a trying experience for most people, especially those working in healthcare at the tip of the spear in combating the COVID-19 pandemic. I am quite certain that the *Journal* will be featuring informative articles on the topic for years to come, and I trust you will enjoy reading the paper in this issue from Fort, Seoane, Unis, and Price-Haywood that explores “Locally Informed Modeling to Predict Hospital and Intensive Care Unit Capacity During the COVID-19 Epidemic.” Of the other 5 original research papers in this issue, one in particular—“Using Multisource Feedback to Assess Resident Communication Skills: Adding a New Dimension to Milestone Data”—is a great read for all medical educators in the graduate medical education realm of the academic continuum, while the editorial by Ly et al promotes greater exposure among the undergraduate medical education/medical school community to a growing specialty. Additionally, consider the manuscript by Tseng, Parrino, and Denton about the use of a cardiopulmonary bypass video as an effective learning tool for medical students during their surgical rotations.

This issue also includes 2 quarterly columns, 2 quality improvement papers, and 11 case reports/clinical observations. Of note among these case reports are 2 papers—“Langerhans Cell Histiocytosis of the Temporal Bone” by Mayer et al and “Spontaneous Pneumothorax in an Elderly Patient With Coronavirus Disease (COVID-19) Pneumonia” by Rehman et al—that detail very unusual cases and offer the reader many teachable moments.

The unprecedented challenges presented by the COVID-19 pandemic and, at the time I am writing this introduction, our now biweekly hurricane threats are testing our individual and collective resilience in the face of crisis. We at Ochsner Health (and other sites around the world) are extremely proud of our care teams who have risen to these challenges with dedication, professionalism, innovation, and flexibility, all while delivering hope to many patients and their families. However, this collective resolve in the face of challenge does not immunize us from the negative impacts of exposure to stress and trauma. Wherever you work and practice, please avail yourself of the options available to you as an employee if you feel the need to seek help or want someone to just talk to.

Referring to the title of this introduction, I was in clinic this past week when Hurricane Sally was rapidly approaching the Gulf South and, for a while, headed directly for the city of New Orleans. Everyone with a computer or smartphone was looking at the track of the storm that appeared to be steadily shifting to the east of us. But Sally was less than 100 miles from the mouth of the mighty Mississippi River, a point way too close for all of us living in New Orleans. The space between the southeastern-most part of Louisiana and the storm was extremely thin, but a space was visible as I perused the weather radar. When I told my nurse that Sally was too close to us for comfort, she responded, “Doc, stop, take a breath, and look at that space once again. That space between us and the storm on radar is the size of the hand of God.” Almost immediately after hearing this, a gentle calmness rushed over me, and then, not much later, the meteorologist on television announced a decided eastern shift in the forecast—away from New Orleans. The sighs of relief from all the members of our healthcare team and the patients were audible throughout our clinic. For just a moment, we experienced a collective joy and peace, knowing we had escaped another named storm once again. We in southeast Louisiana will do our part to assist our friends and neighbors in Alabama, Florida, and Georgia who were most seriously affected by Sally's visit. That's simply what we do; we give as we have so generously received in the past, especially 15 years ago in the aftermath of Hurricane Katrina.

